# De novo design of autocatalytically forming intra- and intermolecular isopeptide bonds to construct rigid covalent protein assemblies

**DOI:** 10.64898/2026.08.13.744004

**Published:** 2026-08-14

**Authors:** Lukas F Milles, Evelyn B Huddy, Ann Carr, Yang Hsia, Xinting Li, Alex Kang, Banumathi Sankaran, Asim K. Bera, David Baker

## Abstract

Isopeptide bonds are amide bonds between amino acid side chains that can form autocatalytically, notably in the pili of Gram-positive bacteria. Here, we design de novo proteins that form both intramolecular and intermolecular isopeptide bonds entirely autocatalytically. We report over 50 designs that form isopeptide bonds, validated by mass spectrometry and 5 crystal structures. We redesign these constructs as split proteins that form a covalent intermolecular isopeptide crosslink when combined. These split designs are orthogonal to the existing isopeptide-based SpyTag/Catcher system, and their formation can be regulated by temperature, providing control over the timing of crosslinking in protein assemblies. We extend these designs to create rigid domain crosslinks that enable the construction of large well ordered symmetric rings of up to 215 kDa that are irreversibly covalently crosslinked by multiple isopeptide bonds into a single molecule. Our results provide insight into the determinants of isopeptide bond formation, considerably expand the set of isopeptide bond crosslinking systems, and establish a framework to construct fully covalent rigid protein assemblies.

## Full Text Availability

The license terms selected by the author(s) for this preprint version do not permit archiving in PMC. The full text is available from the preprint server.

